# Simultaneous Determination of Flavonoids in Different Parts of *Citrus reticulata*‘Chachi’ Fruit by High Performance Liquid Chromatography—Photodiode Array Detection

**DOI:** 10.3390/molecules15085378

**Published:** 2010-08-05

**Authors:** Yinshi Sun, Jianhua Wang, Shubo Gu, Zhengbo Liu, Yujie Zhang, Xiaoxia Zhang

**Affiliations:** 1 State Key Laboratory of Crop Biology, College of Agronomy, Shandong Agricultural University, Taian, 271018, China; 2 Agricultural Science Research Institute of Shandong, Taian, 271000, China

**Keywords:** high performance liquid chromatography–photodiode array detector (HPLC–PDA), quantitative analysis, flavonoids, *Citrus Reticulata*‘Chachi’ fruit, simultaneous determination

## Abstract

Flavonoids are important polyphenolic secondary metabolites in plant. *Citrus reticulata*‘Chachi’ fruit are rich in flavonoids and are being used as functional antioxidant ingredients for the treatment of atherosclerosis and cancer, *etc*. A high performance liquid chromatography-photodiode array detection system was used to analyze five flavonoids, namely, naringin, hesperidin, didymin, tangeretin and nobiletin, in different parts of *C. reticulata*‘Chachi’ fruit. The chromatographic analysis was performed on a C_18_ column with a gradient elution of acetonitrile and water at a flow rate of 1.0 mL/min. Detection was carried out using a photodiode array detector at 280 nm. The calibration curves for the determination of all analytes showed good linearity over the investigated ranges (*R^2^* > 0.9995). Precision and reproducibility were evaluated by six replicated analyses, and the R.S.D. values were less than 0.9% and 2.7%. The recoveries were between 98.37 and 103.89%. This method is promising to improve the quality control of different parts of *C. reticulata*‘Chachi’ fruit.

## 1. Introduction

**Figure 1:**
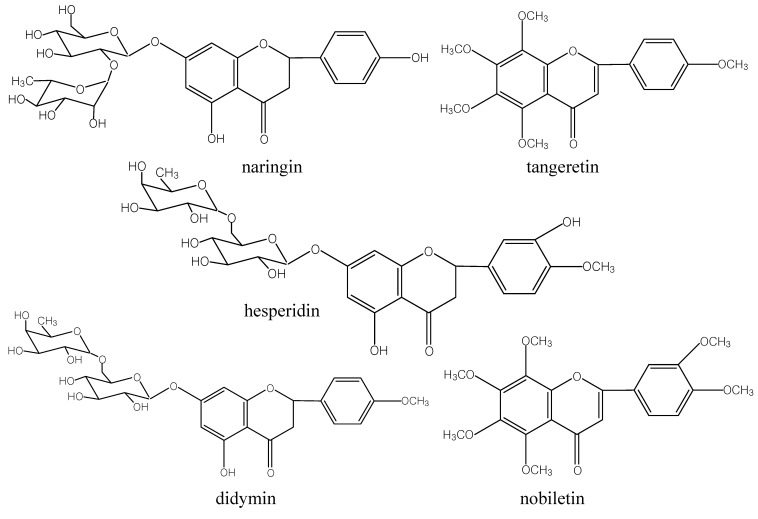
Chemical structures of the five flavonoids.

*Citrus reticulata *‘Chachi’ is a general genus and species (*i.e.* taxon) of tangerines [1,2]. Its dried and mature peel *Pericarpium Citri* Reticultae (Guang Chenpi) has been recorded in the Chinese Pharmacopoeia as appropriate for medical use [3]. *C. reticulata *‘Chachi’ are also consumed as culinary seasonings and tea ingredients in China [4]. Now, pharmacological research has indicated that *Pericarpium Citri *Reticultae exhibits significant antimutagenic [5], antiinflammatory [6,7], antioxidant [8,9], antitumor [10,11], and antiatherosclerosis [12,13] functions and reduces phlegm in the lung [3]. It is well known that various flavonoids including naringin, hesperidin, didymin, tangeretin and nobiletin (structures shown in Figure 1) are the main bioactive constituents of *Pericarpium Citri* Reticultae. Recently, more attention had been paid to flavonoids and some publications have suggested they might play important roles in anticancer activity [14,15,16]. 

So far, quite a few approaches have been developed for the determination of the bioactive constituents from *C. reticulata *‘Chachi’, including many studies on flavonoids from different citrus species and citrus juices [17,18,19,20,21,22], but there is no systematic study on simultaneous determination of five flavonoids in different parts of *C. reticulata*‘Chachi’. The purpose of this work was to determine and analyse five flavonoids in the parts of peel, pith, endocarp, pulp and seeds of *C. reticulata*‘Chachi’ fruit by HPLC, which would be useful for quality control applications to citrus and other plantd associated with these ingredients.

## 2. Results and Discussion

### 2.1. Optimization of the solvent to solid ratio

**Figure 2:**
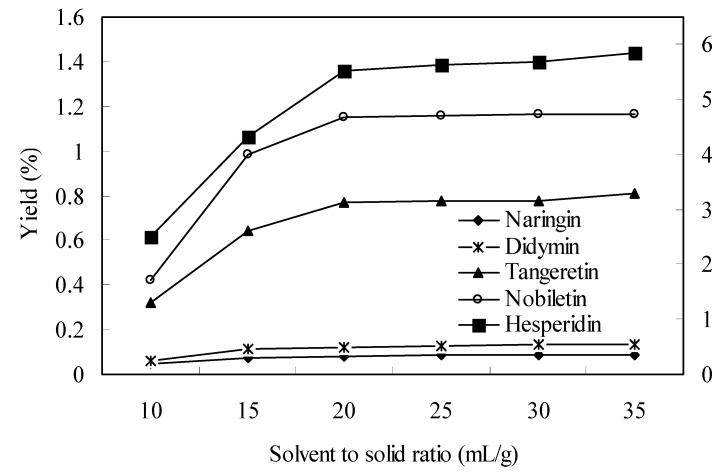
Effect of solvent to solid ratio on the yield of flavonoids (other conditions were fixed at time = 60 min, temperature = 30 °C, power = 250 W).

Generally, a larger solvent volume can dissolve constituents more effectively, leading to an enhancement of the extraction yield [23]. However, this will lead to excess work in the concentration process, and causing a waste of solvent. On the other hand, addition of a small amount of solvent will result in the lower yields of the target constituents [24]. In this study, the solvent to solid ratio was investigated in the range of 10–35 mL/g. As shown in Figure 2, by increasing the solvent to solid ratio, the extraction yields were increased, but when the solvent to solid ratio increased over 20 mL/g, there are no significant differences. Eventually, 20 mL/g was selected as the optimum solvent to solid ratio.

### 2.2. Optimization of the extraction time

**Figure 3:**
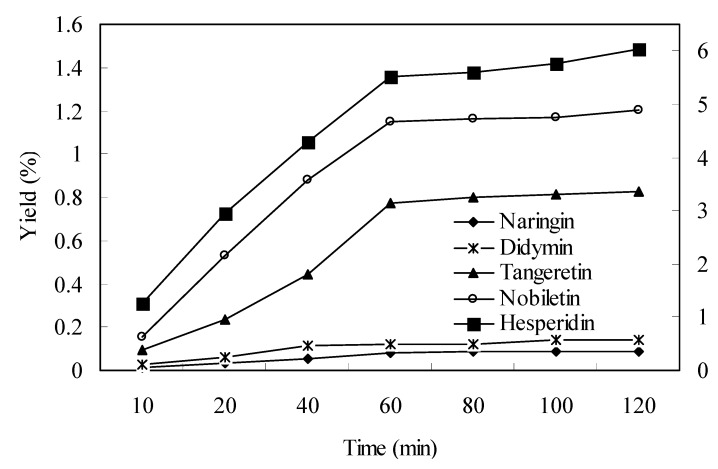
Effect of extraction time on the yield of naringin, hesperidin, didymin, tangeretin and nobiletin (other conditions were fixed at solvent to solid ratio = 20, temperature = 30 °C, power = 250 W).

Figure 3 shows the effects of extraction time on the yield of naringin, hesperidin, didymin, tangeretin and nobiletin. The results indicated that the changing trends of extraction yields of the five target compounds were consistent on the whole. When the extraction time was increased up to 60 min, the increase of the yields were obviously enhanced, but then they leveled off and did not change significantly. Considering that shorter extraction times could cause incomplete extraction and longer extraction timea could be time and solvent wasting, eventually, 60 min was selected as the optimal extraction time.

### 2.3. Optimization of the wavelength

**Figure 4:**
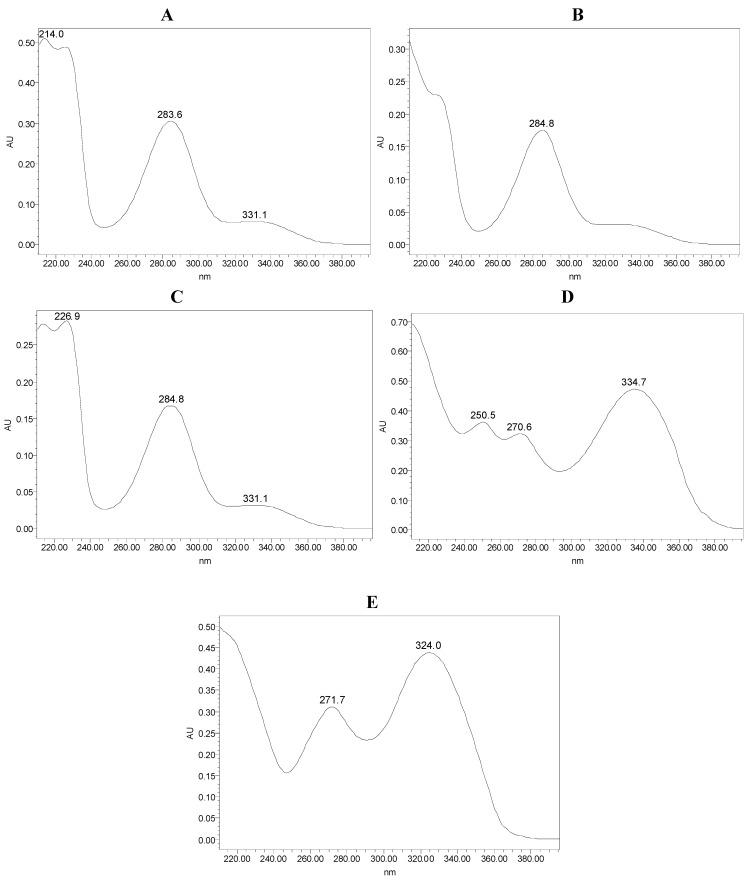
Photodiode array UV spectra: (A) naringin, (B) hesperidin, (C) didymin, (D) tangeretin, (E) nobiletin.

The PDA UV spectra of naringin, hesperidin, didymin, tangeretin and nobiletin were compared in the range of 210–400 nm (Figure 4). The results indicated that naringin (Figure 4A) and hesperidin (Figure 4B) have similar spectra to didymin (Figure 4C), with high absorptions at about 210–227 nm and 283–285 nm. Tangeretin (Figure 4D) showed maximum responses at the wavelengths of 210 nm, 250 nm, 270 nm and 334 nm. Nobiletin (Figure 4E) showed maximum responses at the wavelengths of 210 nm, 271 nm and 324 nm. Although the five components all presented high absorptions in the 210–227 nm range, due to the end absorption of the elution solvent, the baseline of each spectrum was not stationary. Finally, we selected 280 nm as the detection wavelength to eliminate the interference of the elution solvent and to maintain the stability of the determinations.

### 2.4. Optimization of HPLC method

**Figure 5:**
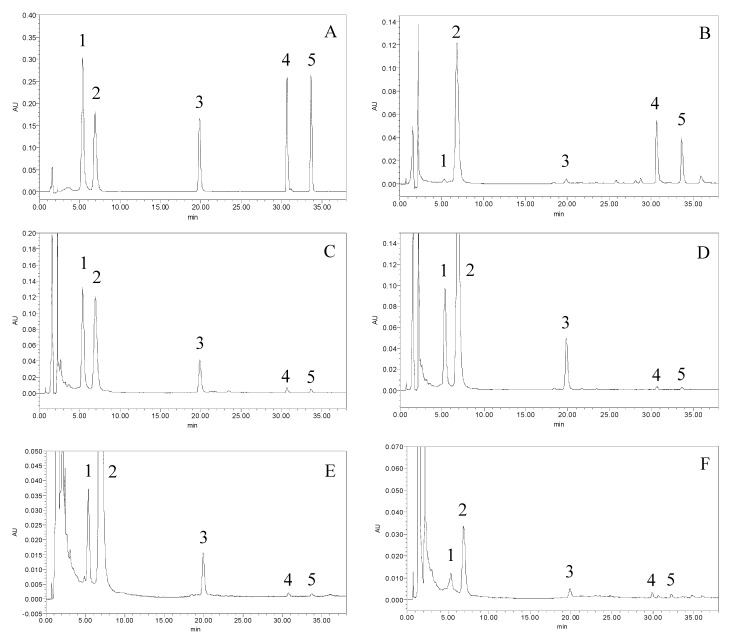
The HPLC chromatograms of the standard mixture solutions and samples. (A) standard mixture solutions, (B) peel, (C) pith, (D) endocarp, (E) pulp, (F) seed. Peaks 1, 2, 3, 4 and 5 correspond to naringin, hesperidin, didymin, tangeretin and nobiletin, respectively.

In the present work, a HPLC method for the analysis of the crude sample was established first. In order to select an appropriate elution system for the HPLC separation of crude samples, different kinds of solvents (methanol–water, acetonitrile–water) were employed to analyze these. The results indicated that when acetonitrile–water was used as the mobile phase, major peaks can be obtained and each peak achieved baseline separation. Besides, the separation conditions of the analytes were optimized by systematically adjusting the acetonitrile content in the mobile phase. Figure 5A shows the standard substances with retention times of 6, 7, 20, 31 and 33 min for naringin, hesperidin, didymin, tangeretin and nobiletin, respectively. Figure 5B–F are the HPLC chromatograms of peel, pith, endocarp, pulp and seeds of *C. reticulata*‘Chachi’ fruit.

### 2.5. Preparation of calibration curve

A series of standard solutions with six different concentrations were analyzed in triplicate by the established method with injection volumes of 20 μL. The calibration curve for each compound was constructed by plotting the peak area (*y*) versus the concentration of standard analyte (*x*, mg/mL). The linear regression equations, correlation coefficients (*R^2^*) and linearity ranges are given in Table 1.

The limit of detection (LOD) and limit of quantification (LOQ) were the concentrations of a compound at which its signal-to-noise ratios (*S/N*) were detected as 3:1 and 10:1, respectively. The values are also given in Table 1.

**Table 1:** Linear regression equation, correlation coefficient and linearity of five flavonoids.

Compound	Regression equation	*R^2^*	Linearity range (μg/mL)	LOD (ng/mL)	LOQ (ng/mL)
naringin	*y* = 212205 *x* - 140.64	0.9996	3.8-121.6	25.3	76.0
hesperidin	*y* = 240262 *x *- 144.7	0.9997	3.1-99.2	20.6	62.0
didymin	*y* = 403272 *x* - 620.05	0.9996	2.0-64.0	13.3	40.0
tangeretin	*y* = 285301 *x* + 40.826	0.9999	2.3-73.6	18.0	53.9
nobiletin	*y* = 345873 *x* + 44.507	0.9999	2.1-67.2	16.4	49.2

### 2.6. Precision

The precision of the method was determined by the analysis of six consecutive injections using the same standard mixture solution. The values of relative standard deviations (R.S.D.s.) of the peak areas for naringin, hesperidin, didymin, tangeretin and nobiletin were 0.9, 0.8, 0.5, 0.3 and 0.7% (*n* = 6), respectively.

### 2.7. Reproducibility test

Reproducibility of peels was evaluated by six replicated analyses. The R.S.D.s. of the contents of naringin, hesperidin, didymin, tangeretin and nobiletin in six replicated peels were 1.5, 2.3, 0.9, 2.7 and 1.4%, respectively (*n* = 6).

### 2.8. Recovery test

In the recovery test, three different concentrations of naringin, hesperidin, didymin, tangeretin and nobiletin were added to known amounts (80, 100 and 120%) of the pre-analyzed peel samples solution, and then the spiked samples were analyzed three times (*n* = 5) by the established HPLC method. The results are shown in Table 2.

**Table 2:** Recovery test of the five flavonoids from peel (*n *= 3).

Components	Quantity added %	Total quantity present (mg)	Amount quantity found (mg)	Recovery (%)	R.S.D.(%)
naringin	80	0.66	0.67	102.02	1.53
	100	0.80	0.82	102.08	0.58
	120	0.95	0.96	101.40	1.53
hesperidin	80	44.22	44.94	101.62	4.16
	100	55.24	54.85	99.29	4.04
	120	66.31	68.43	103.19	1.53
didymin	80	0.97	0.98	101.37	0.58
	100	1.23	1.25	101.36	2.08
	120	1.48	1.47	99.55	0.58
tangeretin	80	6.27	6.38	101.81	4.04
	100	7.75	7.80	100.65	1.00
	120	9.34	9.46	101.28	3.61
nobiletin	80	1.20	1.22	103.89	0.58
	100	1.53	1.56	102.18	2.52
	120	1.84	1.81	98.37	3.00

### 2.9. Application

The optimal conditions were applied to the quantitative analysis of naringin, hesperidin, didymin, tangeretin and nobiletin in different parts of *C. reticulata*‘Chachi’ fruit and the results are presented in Table 3. The chromatograms obtained were shown in Figure 5B–F. The identification of the investigated compounds was carried out by comparison of their retention time and UV spectra of the standard compounds.

**Table 3:** Amount of five flavonoids in *C. reticulata*‘Chachi’ Fruit (*n *= 3).

Analyte	Contents (μg/g) (mean ± S.D.)				
	naringin	hesperidin	didymin	tangeretin	nobiletin
Peel	811.5 ± 18.1	55260.4 ± 802.4	1232.7 ± 21.3	7702.1 ± 80.6	1520.4 ± 40.5
Pith	7083.1 ± 90.7	8538.2 ± 57.6	2228.6 ± 50.8	5.2 ± 0.2	194.2 ± 5.6
Endocarp	3180.2 ± 64.9	1810.8 ± 29.7	1668.6 ± 26.4	1.6 ± 0.0	65.7 ± 2.1
Pulp	584.0 ± 14.2	8369.4 ± 75.1	291.3 ± 7.4	10.7 ± 0.1	17.8 ± 0.3
Seed	79.7 ± 3.3	241.6 ± 9.3	24.9 ± 1.1	3.0 ± 0.1	4.2 ± 0.2

## 3. Experimental

### 3.1. Reagents and materials

Acetonitrile used for HPLC was of chromatographic grade (Yongda Chemical Factory, Tianjin, China), and water used was distilled water. Other organic solvents used were of analytical grade and purchased from Tianjin Chemical Factory (Tianjin, China). A microporous membrane (φ 13 mm, 0.45 μm) from Tianjin Tengda Filtration Instrument Co. (Tianjin, China) was used. Naringin, hesperidin, didymin, tangeretin and nobiletin were obtained from the authors’ laboratory, their structures were fully characterized by chemical and spectroscopic methods (UV, IR, NMR, MS). Their purities were above 98.0% as judged by HPLC–PDA. Fresh *Citrus reticulata*‘Chachi’ fruit was collected from Guangzhou Province, China, and was identified by Dr. Jianhua Wang (College of Agronomy, Shandong Agricultural University).

### 3.2. Instrument and chromatography conditions

The high performance liquid chromatography (HPLC) equipment used was a Waters 600E (USA) system including a 4-solvent delivery system, 600E start-up kit, a 600 pump, 0–20 mL/min, a 2996 photodiode array detector, an Empower Add-on Single System (China), a 4-chamber in-line degasser and a 600E controller. The analysis was performed on a Symmetry C_18_ column (250 mm × 4.6 mm i.d., 5 μm particle size) at ambient room temperature using a gradient elution of acetonitrile (solvent A) and water (solvent B) at a flow rate of 1.0 mL/min. A gradient program was used as follows: 22–22% A at 0–10 min, 22–61% A at 10–35 min and 61–100% A at 35–40 min, re-equilibration duration between two individual runs was 15 min. Detection was carried out at 280 nm.

### 3.3. Sample preparation

The peel, pith, endocarp, pulp, seed of *C. reticulata*‘Chachi’ fruit were separated and dried to a constant weight at 60 °C in a vacuum oven, then pulverized to powder (about 40-mesh) with a disintegrator. The five parts of *C. reticulata*‘Chachi’ fruit ground samples were extracted with methanol (solvent to solid ratio was 20 mL/g) in an ultrasonic water bath for 60 min. All extractions were done at ambient room temperature and each extraction was repeated three times. The extracts were combined and concentrated under reduced pressure. The residue was then reconstituted in methanol to give appropriate concentration. All solutions were filtered through 0.45 μm membrane filter before direct injection into the HPLC system.

### 3.4. Preparation of standard solutions

A mixed standard stock solution was prepared by transferring 3.8 mg/mL of naringin, 3.1 mg/mL of hesperidin, 2.0 mg/mL of didymin, 2.3 mg/mL of tangeretin and 2.1 mg/mL of nobiletin working standards into a 10 mL volumetric flask and making up to the mark with methanol. Then, the mixed solution was diluted step by step with methanol to give six different concentrations of working standard solutions. All solutions were filtered through 0.45 μm membrane filter prior to analysis.

## 4. Conclusions

A simple, accurate and reliable analytical method for the simultaneous determination of five major flavonoids from *C. reticulata*‘Chachi’ fruit by HPLC-PDA has been developed. The flavonoids, naringin, didymin, hesperidin, tangeretin and nobiletin, were determined in different parts of *C. reticulata*‘Chachi’ fruit. The results of the analysis indicated that naringin and didymin were mainly found in pith and endocarp, and hesperidin, tangeretin and nobiletin mainly stored in peel and pith. This method showed good linearity, sensitivity and sufficient limit of detection. The evaluation of data could be useful for screening the optimal condition of extraction of different part of *C. reticulata*‘Chachi’ fruit, and also facilitate to provide the critical quality assurance of related extracts of Chinese medicinal plants. 
